# How do annoying environmental stimuli affect cognitive failures and sleep quality in intensive care unit nurses? Mediating role of mood

**DOI:** 10.17533/udea.iee.v43n2e11

**Published:** 2025-07-25

**Authors:** Zohre Godarzi, Mohammad Sadegh Sohrabi, Rashid Heidarimoghadam, Amin Doosti-Irani, Mohammad Babamiri

**Affiliations:** 1 M.S. Email: bluovay2002@gmail.com. https://orcid.org/0009-0009-0677-3251 Hamadan University of Medical Sciences Iran bluovay2002@gmail.com; 2 . Faculty member, Ph.D. Email: ms.sohrabi@umsha.ac.ir. https://orcid.org/0000-0002-3543-578X Hamadan University of Medical Sciences Iran ms.sohrabi@umsha.ac.ir; 3 . Faculty member, Ph.D. Email: dr_haidari@yahoo.com. https://orcid.org/0000-0002-5711-0150 Hamadan University of Medical Sciences Iran dr_haidari@yahoo.com; 4 . Faculty member, Ph.D. Email: doostiiraniamin@gmail.com. https://orcid.org/0000-0003-0623-7503 Hamadan University of Medical Sciences Iran doostiiraniamin@gmail.com; 5 . Faculty member, Ph.D. Email: mohammad.babamiri@yahoo.com. Corresponding Author. https://orcid.org/0000-0002-0824-8567 Hamadan University of Medical Sciences Iran mohammad.babamiri@yahoo.com; 6 . Department of Ergonomics, School of Public Health, Research Center for Health Sciences, Institute of Health Sciences and Technologies, Hamadan University of Medical Sciences, Hamadan, Iran Hamadan University of Medical Sciences Department of Ergonomics School of Public Health Hamadan University of Medical Sciences Hamadan Iran; 7 . Center of Excellence for Occupational Health, Occupational Health and Safety Research Center, Dept. of Ergonomics, School of Public Health, Hamadan University of Medical Sciences, Hamadan, Iran. Hamadan University of Medical Sciences Center of Excellence for Occupational Health, Occupational Health and Safety Research Center Dept. of Ergonomics, School of Public Health Hamadan University of Medical Sciences Hamadan Iran; 8 . Research Center for Health Sciences, Institute of Health Sciences and Technologies, Avicenna Health Research Institute, Hamadan University of Medical Sciences, Hamadan, Iran Hamadan University of Medical Sciences Research Center for Health Sciences Institute of Health Sciences and Technologies, Avicenna Health Research Institute Hamadan University of Medical Sciences Hamadan Iran; 9 . Department of Epidemiology, School of Public Health, Hamadan University of Medical Sciences, Hamadan, Iran. Hamadan University of Medical Sciences Department of Epidemiology School of Public Health Hamadan University of Medical Sciences Hamadan Iran

**Keywords:** sleep quality, environmental exposure, cognitive dysfunction, affect, nurses, intensive care units, cross-sectional studies., calidad del sueño, exposición a riesgos ambientales, disfunción cognitiva, afecto, enfermeras y enfermeros, unidades de cuidado intensivo, estudios transversales., qualidade do sono, exposição ambiental, disfunção cognitiva, afeto, enfermeiras y enfermeiros, unidades de terapia intensiva, estudos transversais.

## Abstract

**Objective.:**

To investigate the relationship between disturbing environmental stimuli with sleep quality and cognitive failures in intensive care unit nurses, taking into account the mediating role of mood.

**Methods.:**

A cross-sectional study was done with the participation of 201 intensive care unit nurses who were selected by census method from public hospitals in western Iran. Questionnaires were used to collect data, including: pittsburgh sleep quality index (PSQI), occupational cognitive failure questionnaire (OCFQ), Brunel Mood Scale (BRUMS), environmental annoyance and perceived noise annoyance. Modeling was done with univariate linear regression and multivariate regression.

**Results.:**

The results of the study revealed that 84.1% of the participants had poor sleep quality. 13.3% experience cognitive failures at a low level, 61.7% at an average level, and 7% experience cognitive failures at a high level. By examining the mediating role of mood, it was found a significant and negative relationship between positive mood and: annoying environmental stimuli and people's positive mood (p=0.004) and noise annoyance (p=0.002); another significant and negative relationship was also observed between noice annoyance and cognitive failures (p<0.001).

**Conclusion.:**

Considering the mediating role of mood in the effect of environmental variables on cognitive failures and quality of sleep, it is recommended to use psychological interventions to adjust nurses' mood.

## Resumen

**Objetivo.:**

Investigar la relación entre los estímulos ambientales molestos con la calidad del sueño y las fallas en el rendimiento cognitivo en enfermeras de la Unidad de Cuidados Intensivos -UCI-, teniendo en cuenta el papel mediador del estado de ánimo.

**Métodos.:**

Estudio de corte transversal en el que participaron 201 enfermeras de UCIs seleccionadas por el método censal de hospitales públicos del oeste de Irán. Para la recolección de datos se utilizaron 5 instrumentos: Índice de Calidad del Sueño de Pittsburgh (PSQI), Cuestionario de Fracaso Cognitivo Ocupacional (OCFQ), Escala de Estado de Ánimo de Brunel (BRUMS), Molestia ambiental y Molestia por ruido percibido. Se realizaron modelos de regresión lineal univariada y de regresión multivariada.

**Resultados.:**

Los hallazgos del estudio revelaron que el 84.1% de los participantes tenían una mala calidad de sueño. El 13.3% experimenta fallos cognitivos a un nivel bajo, el 61.7% a un nivel medio y el 7% restante a un nivel alto. Al examinar el papel mediador del estado de ánimo, se encontró una relación significativa y negativa entre el estado de ánimo positivo y: los estímulos ambientales molestos y el estado de ánimo positivo de las personas (*p*=0.004), la molestia por ruido (*p*=0.002); también se observó otra relación significativa y negativa entre la molestia por ruido y los fallos cognitivos (*p*<0.001).

**Conclusión.:**

Se encontró que el estado de ánimo tiene un papel mediador en el efecto sobre las variables ambientales, los fallos cognitivos y la calidad del sueño de las enfermeras que laboran en UCIs. Se recomienda utilizar intervenciones para mejorar el estado de ánimo de esta población en este tipo de servicios.

## Resumo

**Objetivo.:**

Investigar a relação entre estímulos ambientais perturbadores e qualidade do sono e comprometimentos cognitivos em enfermeiros de Unidade de Terapia Intensiva (UTI), levando em consideração o papel mediador do humor.

**Métodos.:**

Um estudo transversal envolvendo 201 enfermeiros de UTI selecionados pelo método de censo de hospitais públicos no oeste do Irã. Cinco instrumentos foram usados para coleta de dados: Índice de Qualidade do Sono de Pittsburgh (PSQI), Questionário de Falha Cognitiva Ocupacional (OCFQ), Escala de Humor de Brunel (BRUMS), Incômodo Ambiental e Incômodo de Ruído Percebido. Modelos de regressão linear univariada e regressão multivariada foram realizados.

**Resultados.:**

Os resultados do estudo revelaram que 84.1% dos participantes tinham má qualidade de sono. 13.3% experimentaram lapsos cognitivos em um nível baixo, 61.7% em um nível médio e os 7% restantes em um nível alto. Ao examinar o papel mediador do humor, foi encontrada uma relação significativa e negativa entre humor positivo e: estímulos ambientais irritantes e humor positivo das pessoas (*p*=0.004), incômodo com ruído (*p*=0.002); outra relação significativa e negativa também foi observada entre incômodo com ruído e lapsos cognitivos (*p*<0.001).

**Conclusão.:**

Foi descoberto que o humor tem um papel mediador no efeito sobre variáveis ambientais, lapsos cognitivos e qualidade do sono de enfermeiros que trabalham em UTIs; recomenda-se o uso de intervenções para ajustar o humor dessa população neste tipo de serviço.

## Introduction

Nursing is the most stressful occupation in the healthcare sector, with numerous stressors that can negatively impact the mental health of those employed in this field.^1^. The ability to provide effective nursing care is considered a crucial factor in the recovery of patients in special care units. Among the nursing skills employed in intensive care units is the monitoring of the environment and the creation of a supportive and stress-free atmosphere. This monitoring encompasses the regulation of light, sound, colour, view, music and social sensitivities.^2^. Hospital personnel in a tranquil environment are better equipped to provide medical services to patients . Inappropriate environmental conditions are a significant contributing factor to the creation of mental pressure (tension) that endangers mental health.^3^. The presence of environmental stimuli has been demonstrated to cause destruction and damage to the fundamental physiological needs, and to interfere with the growth, development, and productivity of humans.^4^. A study conducted by Liu et al. in 2023, entitled " Critical indoor environmental factors affecting productivity" identified four environmental factors, including sound, lighting, ventilation, and thermal comfort, that can directly and indirectly influence an individual's mental health and, consequently, their performance and productivity at work.^5^. The response of an individual to a stressor is contingent upon the characteristics of the stimulus and the individual's characteristics. In the absence of a suitable response to a stressor, symptoms such as fatigue, irritability and a lack of concentration may manifest.^4^ A study conducted by G. Belojevic et al. demonstrated that sound has an effect on human cognitive tasks, including memory, alertness, processing, and the accuracy of performance. These effects were observed under the influence of sound. The study revealed that individuals who are particularly sensitive to sound are more susceptible to the adverse effects of noise on cognitive performance.^6^

A study conducted by Kolakari et al. determined that the most common cause of stress among intensive care unit workers is related to unpleasant odours and chemicals. This environmental stimulus can be stressful regardless of causing poisoning .^4^ If the stressors in the work environment are such that the personnel feel unable to meet the existing requirements, adverse physiological and psychological responses will appear, which can endanger the health of the individual and directly affect their physical and mental health and efficiency.^7^ The nursing profession is characterised by shift work and sleep disorders, which may contribute to the prevalence of sleep disorders among nurses and the relationship between human health and sleep quantity and quality.^8^ In addition to the aforementioned consequences, occupational accidents, erroneous decisions and choices are also significant outcomes of suboptimal sleep quality in individuals. These outcomes can be particularly pertinent in professions such as nursing.^9^

Another consequence of environmental stimuli is the impact on individuals' cognitive performance. Indeed, the results of several studies conducted in this field indicate that exposure to environmental stimuli can have detrimental effects on people's cognitive performance, including attention, memory, and reaction time.^10^ Conversely, it appears that one of the areas most affected by sleep quality is the brain capacity and cognitive functions of individuals. In other words, individuals with sleep disorders are likely to be more susceptible to cognitive impairment than those without such disorders.^11^ In addition to sleep quality and cognitive failures, certain internal states and moods can influence how individuals respond to environmental stimuli.^12^ In general, mood states are psychological responses to environmental and periodic stimuli that influence the evaluation and interpretation of psychological situations and the functions of the past, present, and future . The presence of irritating environmental stimuli can result in the manifestation of mental distress, including fatigue, psychological symptoms, and mood fluctuations.^7^^,^^13^ Such mood changes can result in a reduction in the speed of work, an increase in occupational accidents, and a decline in productivity among the organisation's employees . Yari Kia et al. in 2010, it was found that exposure to inappropriate environmental factors such as light, smell, and improper ventilation causes negative mood reactions. Furthermore, it has been demonstrated that inappropriate environmental factors, such as light, smell, and ventilation, can lead to aggressive behaviour in individuals. This study has identified the modification of lighting systems in therapeutic environments as a key strategy for the prevention of negative mood disorders in people.^14^ The objective of the present study is to examine the relationship between exposure to annoying environmental stimuli, sleep quality and cognitive failures in nurses. Additionally, the study will assess the potential for mood mediation in these relationships.

## Methods

This is a cross-sectional study conducted in 2023. The research community comprised intensive care nurses in Borujerd city in Iran. Nurses working in the ICU, CCU, and NICU departments were studied. Sampling was conducted using the census method, and the final sample size was 201 individuals. In order to be eligible for inclusion in the study, participants were required to have at least one year of experience working in intensive care unit. Furthermore, pregnant and lactating women were excluded from the study due to exemption from night shift service. Prior to their participation in the study, all subjects were required to provide informed consent. In addition, they were permitted to withdraw from the study at any point during the research process should they so desire. The data required for the research were collected via five questionnaires, including the questionnaires on pittsburgh sleep quality index (PSQI), occupational cognitive failure questionnaire (OCFQ), Brunel Mood Scale (BRUMS), environmental annoyance and perceived noise annoyance.

### Instruments

*The Pittsburgh Sleep Quality Questionnaire (PSQI)* was utilised to assess the sleep status of the participants. The score of the responses varies from zero to three, based on the grading criteria. The number 3 signifies an unsuitable outcome, while a score of zero (0) indicates a highly suitable result. To assess the quality of sleep, the total score is employed, with a score of 0 to 4 classified as indicative of good sleep quality and a score of 5 or above indicative of poor sleep quality. The questionnaire has been subjected to a validation procedure, which has been conducted by experts in the field. The reliability of this questionnaire was also reported as 0.89 for all questions using the Cronbach's alpha method.^15^

*The Occupational Cognitive Failures Questionnaire (OCFQ*) was employed to assess the aforementioned variable. A 30-item questionnaire (OCFQ) was employed to assess this variable. The grading method employed is a five-point Likert scale, with the options representing a complete disagreement, disagreement, no opinion, agreement, and complete agreement, respectively. The final score is calculated by adding together the total scores for all questions. The resulting score will fall within the range of 30 to 150. A score of 30-60 is indicative of a low level of cognitive error, a score of 60-90 is indicative of a moderate level of cognitive error, and a score above 90 is indicative of a high level of cognitive error. The content validity index (CVI) index of the questionnaire is 0.7, which is within the acceptable range. The reliability of the questionnaire has already been confirmed by other studies.^16^

*The Brunel Mood Scale (BRUMS)* is a psychometric instrument designed to assess mood states. It comprises 32 items, with five-point Likert-type scales, and includes three positive dimensions (calmness, happiness, and vitality) and five negative dimensions (tension, depression, anger, fatigue, and confusion). The questionnaire comprises 32 questions and employs a five-choice Likert-type scale. It encompasses three positive dimensions, namely calmness, happiness and vitality, which collectively account for 12 questions. In contrast, it encompasses five negative dimensions, namely tension, depression, anger, fatigue and confusion, which collectively account for 20 questions. The lowest possible score for an individual on the mood questionnaire is 0, while the highest possible score is 128.^12^ The reliability of the questionnaire was evaluated using Cronbach's alpha by the researchers of the same study, and its value was recorded as 0.86.

*The Environmental Stimuli Questionnaire* was employed to assess the environmental stimuli present within the work environment. The reliability of the questionnaire was evaluated using Cronbach's alpha by the researchers of the same study, and its value was recorded as 0.86. In order to assess the qualitative validity, the questionnaire was initially administered to 10 expert professors specialising in ergonomics. Subsequently, two relative coefficients of content validity, CVR (Content Validity Ratio) and CVI, were employed to assess content validity quantitatively. The CVR and CVI values obtained were 0.78 and 0.93, respectively. Given that the value of CVR and CVI obtained is higher than the minimum value suggested for these coefficients in the relevant standard tables, their values were confirmed. The questionnaire comprises five questions pertaining to both light and smell, which were subjected to both validity and reliability testing. The level of annoyance caused by encountering these two factors is reflected in the responses to the questions. The first option (no) indicates that the respondent is not annoyed by the factor in question. The second option (yes, sometimes) indicates that the respondent is sometimes annoyed by the factor in question. The third option (yes, a lot) indicates that the respondent is frequently annoyed by the factor in question. These responses are then divided into three groups: people annoyed by lighting equipment, people annoyed by smells, and people generally annoyed .

*The Annoying Noise Perception Questionnaire (ANPQ)* is a self-report instrument designed to assess the subjective experience of noise annoyance. The reliability of this questionnaire was assessed using Cronbach's alpha, with a value of 0.82 calculated. In order to assess the qualitative validity of the questionnaire, it was initially administered to 10 expert professors in the field of ergonomics. Subsequently, two relative coefficients of content validity, CVR (Content Validity Ratio) and CVI (Content Validity Index), were employed to assess content validity quantitatively. The values of CVR and CVI obtained for the questionnaire were 0.88 and 0.92, respectively. Given that the values of CVR and CVI obtained are greater than the minimum values suggested for these coefficients in the relevant standard tables, it can be concluded that their values are confirmed. The questionnaire comprises 12 questions and was subjected to a process of validation and reliability testing. The questionnaire comprises both 5-point Likert scale questions and multiple-choice options. The questionnaire comprises subscales pertaining to noise annoyance, mental health (including feelings and symptoms associated with noise), work productivity, adopted solutions against noise, personal strategies against noise, employees' sensitivity and reaction to noise, and an understanding of the source of noise.^17^

### Ethical issues

All participants were aware of the study's objectives and methodology and participated completely voluntarily. The study was also conducted under the supervision of the Ethics Committee of Hamadan University of Medical Sciences with the number IR.UMSHA.REC.1402.098.

### Statistical analysis

This research employed a descriptive and inferential analysis approach, as well as data recording and preliminary investigations utilising the SPSS 23 software suite. Furthermore, confirmatory factor analysis and structural equation modelling were employed to test hypotheses, using STATA software. Finally, univariate and multivariate regression methods with a significance level of 0.05 were applied.

## Results

The participants in this study were 201 nurses, of whom 70.6% were women and 29.4% were men. The age range of the participants was between 24 and 54 years, with an average 34.68 and a standard deviation of 6.36 years. The results obtained from the questionnaire on annoying environmental stimuli revealed that 31 nurses (15.42%) were affected by electrical irritation. It was determined that the electrical sources in the department, including visual alarms, constituted the primary irritating factor. A total of 129 nurses (64.17%) were identified as belonging to the group of individuals whose irritation is affected by chemicals and smells in the ward. The highest score obtained from their questionnaire was related to questions 4 and 5. A total of 44 nurses (21.89%) in the study group exhibited a high level of annoyance, as indicated by their responses to both sets of questions. These questions pertained to electrical irritation and to odours and chemicals.

An examination of the scores obtained from the participants on the noise annoyance questionnaire indicated that 187 individuals (93.1%) exhibited sensitivity and annoyance in response to noise, whereas only 14 individuals (6.9%) were affected by noise and noise-related distress in the intensive care unit. Among the mental symptoms caused by people being exposed to noise, the most frequently reported feelings are related to lack of concentration during the task and dissatisfaction. These feelings were experienced by 38.30 and 19.40% of people, respectively. Loss of motivation was reported in only one study subject. Among the sound sources in the special care department, the most frequent complaints of patients are due to continuous exposure to alarms related to medical equipment (ventilator, wavy mattresses, etc.) and the voices of other patients and staff. These were reported by 43.3 and 35.8 percent of patients, respectively. The least frequent source of complaint among patients was noise from sirens and alarms, which was reported in only 2.5% of cases. A total of 164 (82%) of the study participants demonstrated sensitivity to the noise of their work environment, exhibiting a range of reactions. In contrast, 38 (19%) of them did not exhibit any specific reaction to the noise of their environment. The noise in the intensive care unit had an adverse effect on the performance and productivity of 163 participants (81%), while 38 nurses (18%) did not feel any change in their work productivity under the influence of this factor. Among the 201 individuals under study, 79 (39.3%) opted to request that others reduce their voice levels in order to mitigate the impact of noise in the workplace. Only 1% of respondents indicated that they had adopted a different task to address this factor.

**Figure 1:**
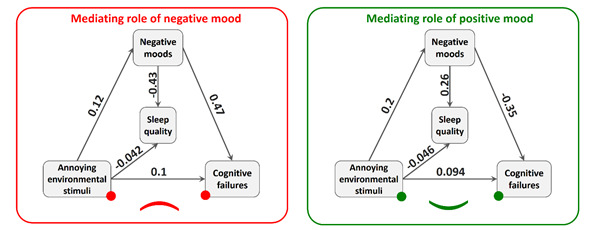
The relationship between annoying environmental stimuli, sleep quality and cognitive failures; mediating role of positive mood (right) and negative mood (left)

The findings of the sleep quality questionnaire indicated that 32 individuals (15.9%) exhibited good sleep quality, while 169 individuals (84.1%) demonstrated poor sleep quality. Additionally, the analysis revealed that there was no significant correlation between annoying environmental stimuli and sleep quality. These relationships are depicted in Figure 1. However, a negative and significant relationship was observed between noise annoyance and sleep quality (see Table 1). Among the participants, 63 individuals (13.3%) exhibited low-level cognitive failures, 124 (61.7%) exhibited average-level cognitive failures, and 14 (7%) exhibited high-level cognitive failures. A significant relationship was observed between noise annoyance and cognitive failures of people (*p* < 0.0001), but no significant relationship was observed between annoying environmental stimuli and cognitive failures of people (*p* = 0.048).

**Table 1:** Linear relationships between dependent and independent variables

Dependent variables	Independent variables	Coefficient	***p-*value**	Lower limit	Upper limit
Cognitive failure	Environmental irritants	0.70	0.048	0.00	1.40
	Voice annoyance	1.25	<0.001	0.89	1.60
Sleep quality	Environmental irritants	-0.29	0.046	-0.59	-0.004
	Noise annoyance	-0.32	<0.001	-0.48	-0.16
Positive mood	Noise annoyance	-0.30	0.003	-0.50	-0.10
	Environmental irritants	-0.22	0.235	-0.59	0.14
Negative mood	Environmental irritants	1.15	<0.001	0.60	1.70
	Noise annoyance	0.64	<0.001	0.33	0.94
Cognitive failure	Environmental irritants	0.70	0.048	0.00	1.40
	Noise annoyance	1.25	<0.001	0.89	1.60
Sleep quality	Environmental irritants	-0.29	0.046	-0.59	-0.004
	Noise annoyance	-0.32	<0.001	-0.48	-0.16
Positive mood	Noise annoyance	-0.30	0.003	-0.50	-0.10
	Environmental irritants	-0.22	0.235	-0.59	0.14
Negative mood	Environmental irritants	1.15	<0.001	0.60	1.70
	Noise annoyance	0.64	<0.001	0.33	0.94

By examining the mediating role of mood, it was found that there is a significant relationship between annoying environmental stimuli and people's positive mood (*p*=0.004). This indicates that with the increase in the amount of annoying environmental stimuli, the positive mood of people decreases. However, between annoying environmental stimuli and negative mood, there is no significant relationship (*p*=0.073) (Table 2). Furthermore, the results indicate that there is a relationship between noise annoyance and sleep quality, with the mediating role of positive and negative mood in intensive care nurses (see Table 3).

**Table 2:** The relationship between annoying environmental stimuli and sleep quality: the mediating role of positive and negative mood

Dependent variables	Independent variables	Coefficient	***p*-value**	Lower limit	Upper limit
Positive mood	Irritating environmental stimuli	-0.19	0.004	-0.32	-0/06
Negative mood	Irritating environmental stimuli	0.12	0.073	-0.01	0.25
	Positive moods	0.25	0.000	0.12	0.38
	Irritating environmental stimuli	-0.04	0.509	0.18	0.08
Sleep quality	Negative moods	-0.43	0.000	-0/54	-0.31
	Irritating environmental stimuli	-0.04	0.507	-0/16	0.08
Variance	Sleep quality	0.92	-	0.86	0.99
	Positive moods	0.96	-	0.91	1.01
	Sleep quality	0.80	-	0.71	0.91
	Negative moods	0.98	-	0.95	1.01

Statistical fit test. TLI: 0.000; CFI: 1.000; RMSE: 0.000.

**Table 3:** The relationship between noise annoyance and sleep quality: the mediating role of positive and negative mood

Dependent variables	Independent variables	Coefficient	***p*-value**	Lower limit	Upper limit
Positive mood	Noise annoyance	-0.20	0.002	-0.33	-0/07
Negative mood	Noise annoyance	0.27	<0.001	0.15	0.40
	Positive moods	0.21	0.001	0.08	0.34
	Noise annoyance	0.23	<0.001	-0.35	-0.10
Sleep quality	Negative moods	-0.38	0.000	-0/50	-0.27
	Noise annoyance	-0.16	<0.001	-0/29	-0.04
Variance	Sleep quality	0.87	-	0.79	0.96
	Positive moods	0.95	-	0.90	1.01
	Sleep quality	0.78	-	0.69	0.89
	Negative moods	0.92	-	0.85	1.99

Statistical fit test. TLI: 0.000; CFI: 1.000; RMSE: 0.000.

**Figure 2:**
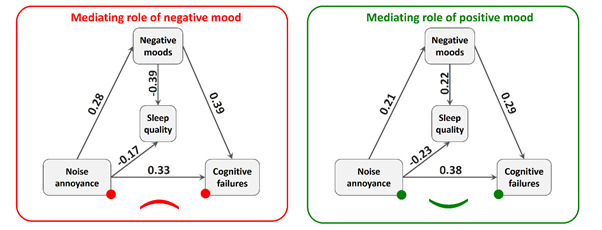
The relationship between noise annoyance, sleep quality and cognitive failures; mediating role of positive mood (right) and negative mood (left)

Furthermore, the results indicate that there is a relationship between annoying environmental stimuli and cognitive failures, with the mediating role of positive mood (*p*=0.004). However, there is no relationship with negative mood (*p*=0.087). The specifics of these interconnections are showed in Table 4. A significant correlation was observed between noise annoyance and positive mood (*p*=0.002), indicating that as noise annoyance increases, positive mood declines. Additionally, there is a significant correlation between noise annoyance and negative mood (*p*<0.001), indicating that an increase in noise annoyance is associated with an increase in negative mood. The data presented in Table 5 indicates a correlation between noise annoyance and cognitive failures, with the mediation of positive and negative mood in intensive care nurses. These relationships are depicted in Figure 2.

**Table 4:** The relationship between annoying environmental stimuli and cognitive failures: the mediating role of positive and negative mood

Dependent variables	Independent variables	Coefficient	***p*-value**	Lower limit	Upper limit
Positive mood	Irritating environmental stimuli	-0.19	0.004	-0.32	-0/06
Negative mood	Irritating environmental stimuli	0.12	0.073	-0.01	0.25
Cognitive failure	Positive moods	-0.35	<0.001	-0.47	-0.23
	Irritating environmental stimuli	0.09	0.155	-0.03	0.22
	Negative moods	0.47	<0.001	0.36	0.57
	Irritating environmental stimuli	0.10	0.087	-0/01	0.22
	Positive moods	0.96	-	0.91	1.01
Variance	Cognitive failure	0.85	-	0.76	0.94
	Negative moods	0.98	-	0.95	1.01
	Cognitive failure	0.75	-	0.65	0.86

Statistical fit test. TLI: 0.000; CFI: 1.000; RMSE: 0.000.

**Table 5:** The relationship between noise annoyance and cognitive failures: the mediating role of positive and negative mood

Dependent variables	Independent variables	Coefficient	***p*-value**	Lower limit	Upper limit
Positive mood	Noise annoyance	-0.20	0.002	-0.33	-0/07
Negative mood	Noise annoyance	0.27	<0.001	0.15	0.40
Cognitive failure	Positive moods	-0.29	<0.001	-0.40	-0.17
	Noise annoyance	0.37	<0.001	0.27	0.48
	Negative moods	0.39	<0.001	0.28	0.50
	Noise annoyance	0.33	<0.001	0.22	0.44
	Positive moods	0.95	-	0.90	1.01
	Cognitive failure	0.66	-	0.56	0.77
	Negative moods	0.92	-	0.85	0.99

Statistical fit test. TLI: 0.000; CFI: 1.000; RMSE: 0.000.

## Discussion

The present study was conducted with the aim of examining the relationship between annoying environmental stimuli and cognitive failures, alongside sleep quality in intensive care unit nurses, with mood as the mediating factor. The findings indicated that the quality of sleep was directly correlated with the exposure to noise in the work environment. However, no significant association was observed between the smell and lighting of the environment and the quality of sleep. Among the effects of chronic exposure to noise, we can cite the primary consequences of delayed sleep onset, sleep fragmentation, and shortened sleep duration . The findings of the present study indicate that the most significant source of noise annoyance in intensive care units is the sound generated by medical equipment and the human voice. This includes staff conversations, expressions of pain by patients, and the voices of patients' companions. The study by Nasari *et al.*^18^ revealed that numerous environmental factors, including light and sound, in intensive care units can induce stress and sleep disorders in patients. Furthermore, it was found that 17-57% of sleep disorders in these units are attributable to the sound alarms of the equipment. In addition, the study revealed that 25% of patients received calls from the department. Furthermore, the study conducted by Waye and colleagues demonstrated that approximately 11-17% of the sleep disturbances experienced by healthcare professionals in intensive care units are attributable to noise. According to the findings of this study, the primary source of auditory interference was medical equipment alarms and human-produced noises .^19^ In a related study, Freedman asserted that noise-induced sleep disturbances can be mitigated in intensive care units by eliminating frequent noise exposures, when compared to other sleep disruptors.^20^

The findings of this study indicate that there is no correlation between the lighting of the workplace and the exposure to chemical odours and the incidence of cognitive failures. However, there is a significant and direct relationship between the exposure to noise in the workplace and the incidence of cognitive failures in special care units. Additionally, there is no significant relationship between qualitative variables, including age and gender, and the incidence of these failures. In their study, Jinjing, Jing and Xiaowei also demonstrated that environmental noise has a detrimental effect on work performance, health and safety. The findings indicated a negative correlation between the quality of the participants' performance and their exposure to sound. This was evidenced by the observation that exposure to sound resulted in alterations to individuals' internal cognitive states, including changes in attention, stress levels, and mental workload.^7^^,^^21^

Neil Ellis's research also revealed that continuous exposure to sound has a detrimental effect on people's level of alertness and attention, increasing the likelihood of performance errors and fatigue.^22^ Yuyan Chen *et al.* demonstrated that environmental conditions, including ventilation in work environments, influence cognitive performance, with a negative correlation between the two variables.^23^ Schiavon *et al.* demonstrated that environmental conditions, specifically lighting, influence cognitive performance, resulting in increased positive emotions, decreased negative emotions, enhanced work memory, and improved concentration.^24^

In addition to sleep quality and cognitive failures, certain internal states and moods can also influence how individuals respond to environmental stimuli.^12^ In general, mood states are a psychological response to an environmental and periodic stimulus that intervenes in the evaluation and interpretation of a psychological situation and how to react to it. The results of this research indicate that annoying stimuli (light and smell) have no effect on the fluctuation of positive mood dimensions (vitality, happiness, calmness).^12^ However, they do appear to increase the level of negative mood dimensions (tension, confusion, fatigue, depression, anger). Conversely, noise exposure in intensive care units has been found to reduce the level of positive dimensions and increase the level of negative dimensions. The findings of this study indicate that mood does not act as a mediator in the effect of odor and light stimuli on cognitive failures and sleep quality. However, it does play a mediating role in the effect of sound on the occurrence of cognitive failures and sleep quality. The study by Passos *et al.* also demonstrated that noise exposure can influence mood and performance, resulting in fatigue and irritability.^25^

It should be noted that every research study, including its valuable findings and results, is also accompanied by limitations and weaknesses. One limitation of this study is that data collection was conducted exclusively through questionnaire methods, which is the most accessible approach for establishing communication with personnel working in these departments. This was due to the sensitivity of special care units and the need for constant monitoring of patients during their hospitalisation period in these units. Additionally, the lack of examination of the variables separately for different work shifts (morning, evening, night) suggests that future studies should investigate the effect of this factor.

Conclusion. Given the significant impact of noise exposure on sleep quality variables, such as sleep efficiency, and cognitive performance and mood, this study suggests a series of control strategies to mitigate noise events. These include eliminating the sound of doors opening and closing, reducing the level of the loudest alarms, separating the call answering room by phone, asking people to reduce their conversation volume, and communicating with patients' companions through short message sending systems instead of phone calls. Moreover, it is acknowledged that environmental stimuli can influence sleep quality and cognitive performance. This can be attributed to the impact of mood on these outcomes. Therefore, it is crucial to consider the role of psychological interventions in regulating mood, as this can effectively reduce the impact of environmental stressors. These interventions include reinterpretation methods, which alter one's view of a situation, self-soothing methods, attention control methods, acceptance methods, and problem-solving abilities. By addressing mood, these approaches can help mitigate the effects of environmental stressors on sleep quality and cognitive performance.
